# Human skeletal myopathy myosin mutations disrupt myosin head sequestration

**DOI:** 10.1172/jci.insight.172322

**Published:** 2023-11-08

**Authors:** Glenn Carrington, Abbi Hau, Sarah Kosta, Hannah F. Dugdale, Francesco Muntoni, Adele D’Amico, Peter Van den Bergh, Norma B. Romero, Edoardo Malfatti, Juan Jesus Vilchez, Anders Oldfors, Sander Pajusalu, Katrin Õunap, Marta Giralt-Pujol, Edmar Zanoteli, Kenneth S. Campbell, Hiroyuki Iwamoto, Michelle Peckham, Julien Ochala

**Affiliations:** 1 The Astbury Centre for Structural and Molecular Biology and; 2 School of Molecular and Cellular Biology, Faculty of Biological Sciences, University of Leeds, Leeds, United Kingdom.; 3 Centre of Human and Applied Physiological Sciences and; 4 Randall Centre for Cell and Molecular Biophysics, School of Basic & Medical Biosciences, Faculty of Life Sciences & Medicine, King’s College London, United Kingdom.; 5 Department of Physiology, University of Kentucky, Lexington, Kentucky, USA.; 6 School of Sport, Exercise and Health Sciences, Loughborough University, Loughborough, United Kingdom.; 7 UCL Great Ormond Street Institute of Child Health, London, United Kingdom.; 8 NIHR Biomedical Research Centre at Great Ormond Street Hospital, Great Ormond Street, London, United Kingdom.; 9 Department of Neurosciences, Unit of Neuromuscular and Neurodegenerative Disorders, IRCCS Bambino Gesù Children’s Hospital, Rome, Italy.; 10 Neuromuscular Reference Center, Neurology Department, University Hospital Saint-Luc, Brussels, Belgium.; 11 Neuromuscular Morphology Unit, Institute of Myology, Myology Research Centre INSERM, Sorbonne University, Hôpital Pitié-Salpêtrière, Paris, France.; 12 APHP, Centre de Référence de Pathologie Neuromusculaire Nord-Est-Ile-de-France, Henri Mondor Hospital, Inserm U955, Creteil, France.; 13 U1179 UVSQ-INSERM Handicap Neuromuscular: Physiology, Biotherapy and Applied Pharmacology, UFR Simone Veil-Santé, Université Versailles Saint Quentin en Yvelines, Paris-Saclay, France.; 14 Neuromuscular and Ataxias Research Group, Instituto de Investigación Sanitaria La Fe, Valencia, Spain.; 15 Centro de Investigación Biomédica en Red de Enfermedades Raras (CIBERER) Spain, Valencia, Spain.; 16 Department of Laboratory Medicine, University of Gothenburg, Gothenburg, Sweden.; 17 Genetics and Personalized Medicine Clinic, Tartu University Hospital, Tartu, Estonia.; 18 Department of Clinical Genetics, Institute of Clinical Medicine, University of Tartu, Tartu, Estonia.; 19 Universidade de São Paulo, Hospital das Clínicas, Faculty of Medicine, Department of Neurology, São Paulo SP, Brazil.; 20 Universidade Federal de São Paulo, Escola Paulista de Medicina, Department of Neurology, São Paulo SP, Brazil.; 21 Division of Cardiovascular Medicine, University of Kentucky, Lexington, Kentucky, USA.; 22 SPring-8, Japan Synchrotron Radiation Research Institute, Hyogo, Japan.; 23 Department of Biomedical Sciences, University of Copenhagen, Copenhagen, Denmark.

**Keywords:** Muscle Biology, Genetic diseases, Neuromuscular disease

## Abstract

Myosin heavy chains encoded by *MYH7* and *MYH2* are abundant in human skeletal muscle and important for muscle contraction. However, it is unclear how mutations in these genes disrupt myosin structure and function leading to skeletal muscle myopathies termed myosinopathies. Here, we used multiple approaches to analyze the effects of common *MYH7* and *MYH2* mutations in the light meromyosin (LMM) region of myosin. Analyses of expressed and purified *MYH7* and *MYH2* LMM mutant proteins combined with in silico modeling showed that myosin coiled coil structure and packing of filaments in vitro are commonly disrupted. Using muscle biopsies from patients and fluorescent ATP analog chase protocols to estimate the proportion of myosin heads that were super-relaxed, together with x-ray diffraction measurements to estimate myosin head order, we found that basal myosin ATP consumption was increased and the myosin super-relaxed state was decreased in vivo. In addition, myofiber mechanics experiments to investigate contractile function showed that myofiber contractility was not affected. These findings indicate that the structural remodeling associated with LMM mutations induces a pathogenic state in which formation of shutdown heads is impaired, thus increasing myosin head ATP demand in the filaments, rather than affecting contractility. These key findings will help design future therapies for myosinopathies.

## Graphical abstract

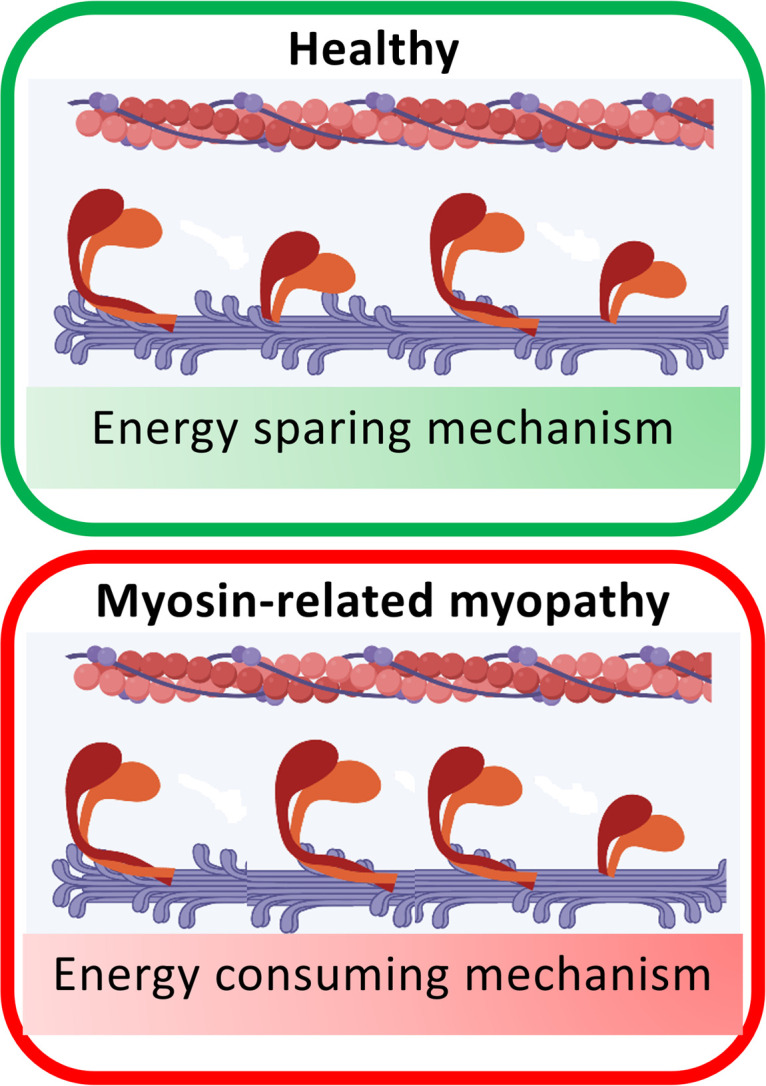

## Introduction

**Figure 1:**
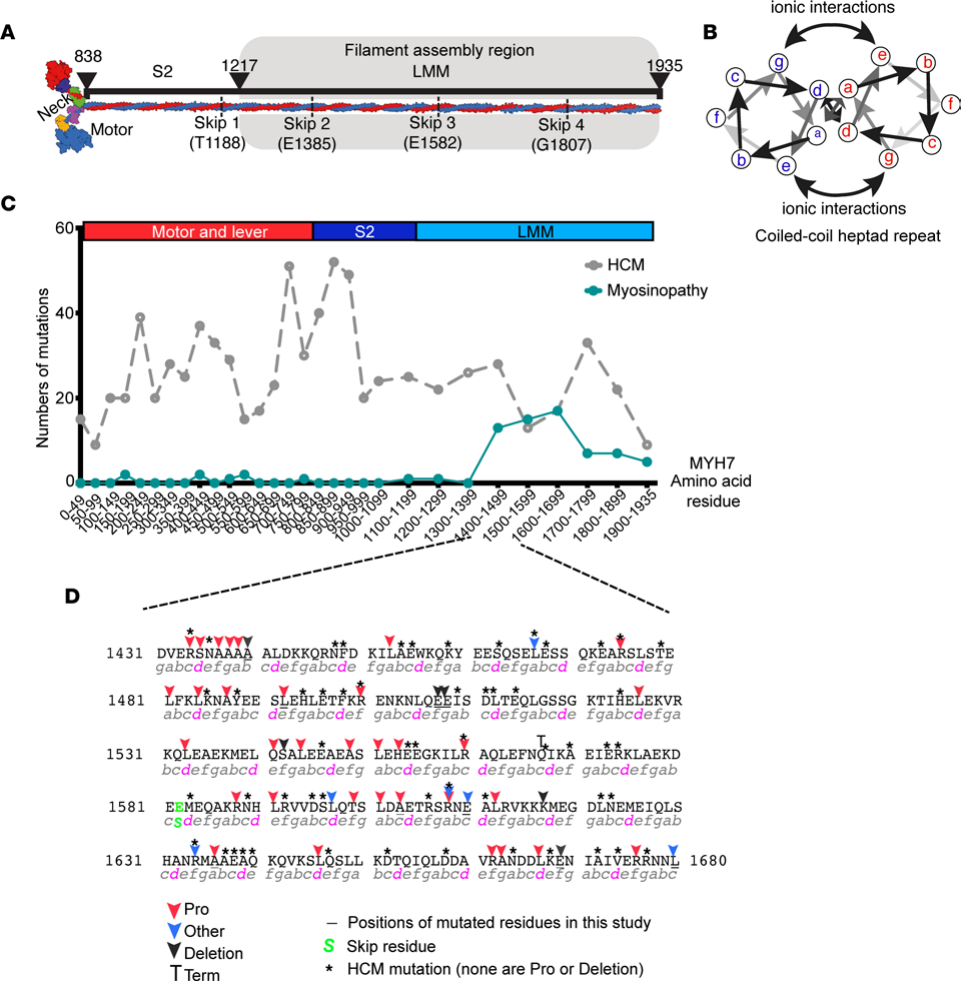
Myosinopathies and location of mutations. (**A**) A schematic showing the overall composition of striated myosin. The molecule is formed by 2 heavy chains that dimerize to form a coiled coil tail composed of subfragment-2 (S-2) and light-meromyosin (LMM). The 2 heavy chains diverge to form the neck of lever, to which light chains bind, and the 2 motor domains, which bind actin and nucleotide. (**B**) A schematic showing an end on view of the heptad repeat of 2 interacting α-helices. Residues in “a” and “d” positions form the hydrophobic seam. (**C**) The frequency of mutations in MYH7 for hypertrophic cardiomyopathy (HCM) (gray dotted line) and for skeletal myopathies (myosinopathy, green line), across the amino acid sequence of MYH7. (**D**) The sequence in which mutations that cause myosinopathies are most frequent. Positions of mutations (commonly mutation to proline or a single amino acid deletion) are indicated by colored arrows. Mutations in residues mutated in HCM are indicated by an asterisk. Underlined residues indicate the position of mutated residues studied here. The heptad repeat is shown underneath the sequence, with “d” positions highlighted in magenta.

Myosin, organized into thick filaments in skeletal and cardiac muscle, interacts with actin in the thin filaments to generate contraction and shortening. Muscle myosins are composed of 2 heavy chains and 2 pairs of light chains (1). The first 838 residues (N-terminal region) form the myosin head, composed of the motor and light chain binding domain (Figure 1A). The motor domain interacts with actin and hydrolyzes ATP. The light chain binding domain contains 2 IQ (isoleucine glutamine) repeats, of which the first binds essential light chain, and the second binds regulatory light chain (1). The downstream C-terminal sequence, starting after the invariant proline residue at position 838 (~60%), forms an α-helix that dimerizes with a second myosin heavy chain (MHC) to form an α-helical coiled coil tail (Figure 1B). The first third of the tail is known as subfragment-2 (S2), and the remaining C-terminal two-thirds is known as light meromyosin (LMM).

The LMM region of the myosin tail directs myosin assembly into filaments via clusters of alternating positive and negatively charged residues, each 28 residues long (42 Å) (2). This results in a quasihelical array of myosin heads on the surface of the filament, in which 3 pairs or heads protrude from the filament every 14.3 Å, forming a “crown” of heads (3). This repeating feature gives rise to the M3 layer line in x-ray diffraction. The helical repeat, comprising 3 crowns, is 430 Å and is equivalent to the axial stagger between adjacent LMM molecules in the thick filament. Two recent CryoEM structures of the C-zone (myosin-binding protein-C–containing region) of cardiac muscle in relaxing conditions, have revealed the complex packing of the myosin tails (S2 and LMM) into the thick filament, together with the interactions of both myosin-binding protein C (MyBPC) and titin with myosin in the filament but not with each other (4, 5).

Until recently, striated muscle contraction was thought to be solely regulated by the binding of Ca^2+^ to thin-filament proteins, shifting the position of tropomyosin on actin to reveal sites to which myosin prefers to bind (6). It has now become clear that activation of myosin heads, particularly in the C-zone of the thick filament, is also important. In relaxed muscle, the myosin heads adopt a shutdown state in which the 2 heads interact with each other and with S2 to form an interacting heads motif (IHM) (7). This led to the idea that the heads need to be released from this state to drive contraction (8, 9). X-ray diffraction approaches have demonstrated that this release is indeed an important component of muscle contraction (10–12). Moreover, this shutdown state is likely to be linked to the super-relaxed state (SRX) of myosin (8, 9) in which the ATPase rate is approximately 5- to 10-fold lower than that of disordered-relaxed (DRX) myosin heads (8, 13). The adoption of the SRX state is likely to be important in conserving energy between muscle contractions (8).

The key role of the SRX state in striated muscle has driven new concepts about how some mutations in myosin, and other striated muscle proteins, might lead to striated muscle disease. A dominant missense mutation in the MHC gene, *MYH7*, was first identified many years ago to cause hypertrophic cardiomyopathy (14). *MYH7* encodes the β/slow MHC, which is expressed in both the heart and in slow skeletal muscle fibers. Over 1,000 missense mutations have since been described for *MYH7*, most of which are dominant, and these have now been implicated in several forms of heart disease (15). Approximately 50% of the known mutations are found in the myosin head, 20% in S2, and the remainder in LMM. While some mutations in the head are likely to directly affect the interaction with actin, others have been suggested to disrupt the IHM, shifting myosin heads into the DRX state and thus increasing ATP consumption and metabolic demands on the muscle (9). Indeed, mavacamten, thought to promote the SRX state (16) has recently been approved as a drug to treat hypertrophic cardiomyopathy (17).

While the effect of mutations in the myosin head are becoming clearer, we still lack a clear understanding as to how the mutations in LMM result in muscle diseases. A subset of these mutations (about 70) in *MYH7* primarily result in skeletal muscle diseases known as myosinopathies rather than cardiomyopathies (18). The overall prevalence of myosinopathies is thought to be 1:26,000 (18), but this number is likely to be an underestimate because of underdiagnosis (19, 20). Strikingly, almost all of these mutations are located in LMM (15). In addition, over 20 dominant/recessive mutations have recently been reported for *MYH2,* which encodes fast skeletal myosin heavy-chain 2A, of which at least 5 are present in LMM (15).

The molecular and cellular mechanisms by which specific mutations in LMM lead to myosinopathies remain unclear and need addressing. We recently showed that 2 of these mutations, A1603P and K1617del, disrupt the structure of the coiled coil and/or myosin filament formation (21) using a combination of in vitro and cellular assays. While LMM is not directly involved in stabilizing the SRX state of myosin, disruption of myosin packing within the thick filament, with potential effects on titin and MyBPC organization, could indirectly induce a decrease in heads in the SRX state, increasing ATP usage in skeletal muscle. Hence, in the present study, we performed a comprehensive analysis of several mutations in *MYH7* and *MYH2*, implicated in myosinopathies. We used circular dichroism and in silico modeling to determine the effects of mutations on secondary structure of LMM, EM to determine effects on LMM filament assembly, GFP-tagged myosin constructs to determine filament formation in cultured muscle cells, Mant-ATP assays to determine the ratio of SRX/DRX heads in samples from human tissue, and force and x-ray diffraction measurements of human tissue samples to gain a better understanding of how mutations in LMM cause myosinopathies.

## Results

### Mutations that cause myosinopathies are clustered in LMM.

An analysis of mutations in *MYH7* that result in hypertrophic cardiomyopathy (HCM) and myosinopathies shows that mutations that cause HCM are distributed throughout the molecule, whereas those causing myosinopathies are mainly restricted to a specific region within LMM, between residues 1400 and 1700 (Figure 1C). A closer look at the types of mutation that cause myosinopathies shows that they are mainly composed of mutations to proline (red arrows, Figure 1D). The next most common type of mutation is a deletion of a single residue, while mutations to other residues are least common (Figure 1D). In contrast, mutations that cause HCM, also found in this region, typically occur in different residues, are all missense mutations excluding mutation to proline, or are single-residue deletions (6). Proline residues are not usually found in coiled coils, since the kink that they introduce into the α-helix is typically not compatible with coiled coil formation. Deletion of a single residue introduces a shift in the heptad repeat pattern and is also likely to introduce a local disruption within the coiled coil. Thus, both types of mutation, found in myosinopathies, are likely to disrupt the secondary structure of the coiled coil, which in turn could have effects on packing of LMM into filaments.

### LMM mutations alter the secondary structure of the coiled coil.

To determine the effects of specific mutations in LMM that result in myosinopathies, we measured the circular dichroism (CD) spectra of expressed and purified LMM constructs for MYH7 and MYH2. The constructs are expressed and purified as GST-LMM proteins, in which the 2 α-helices have already formed a coiled coil during expression in *E*. *coli*; thus, each of the α-helices contains the mutation. The GST is cleaved off prior to carrying out the CD experiments.

**Figure 2:**
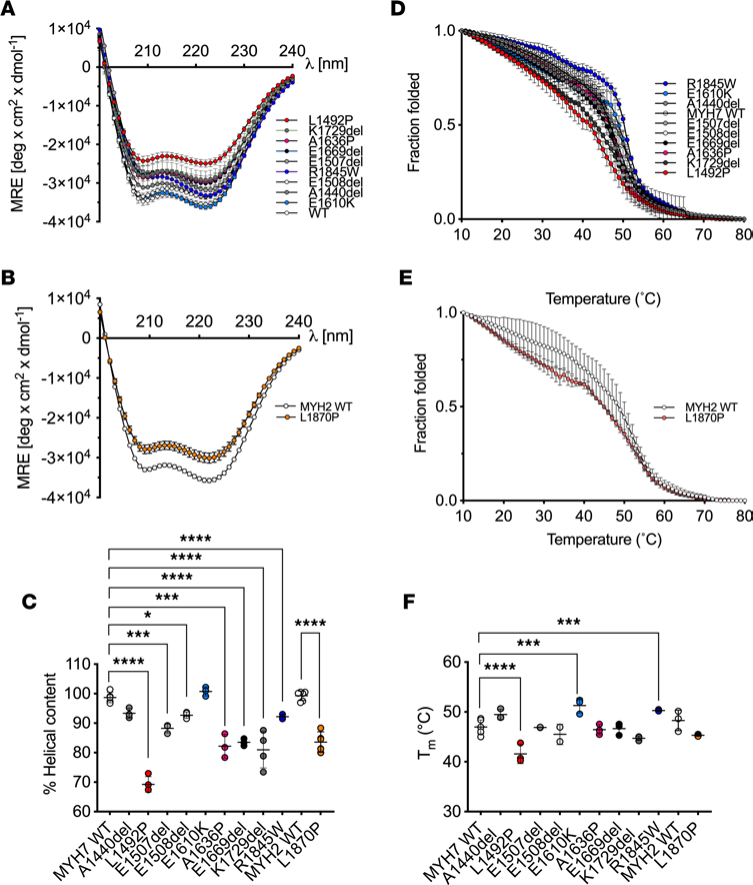
Secondary structure of LMM constructs. (**A** and **B**) The mean circular dichroism (CD) spectra at 10°C for each of the MYH7 (**A**) and MYH2 (**B**) LMM constructs. Data are shown as mean ± SD. MRE, mean residue ellipticity. Mutations to proline are shown in shades of red, deletion mutations in shades of gray, and other mutations in shades of blue. (**C**) The percentage of helicity for each MYH7 and MYH2 LMM construct calculated from the 222 nm MRE values from a minimum of 3 separate experiments. Individual values together with the mean ± SD are shown. The normalized MRE measured at 222 nm from 10°C to 70°C for MYH7 (**D**) and MYH2 (**E**) constructs for a minimum of 3 experiments. These data were used to calculate T_m_ (temperature at which half the protein is melted) for each construct plotted in **F**. Individual data points for each measurement, together with the mean values ± SD, are shown. Significant differences compared with WT are indicated; the 1-way ANOVA with Dunnett’s test post hoc correction was used with *P < 0.05; ***P < 0.001; ****P < 0.0001. Heptad positions for the mutated residues in MYH7: A1440 and A1636 ‘*b*’; E1610 and K1729 ‘*c*’; L1492 ‘*e*’; E1507 and R1845 ‘*f*’; E1508 ‘*g*’ and in MYH2: L1870 ‘*d*’.

These data show that all the constructs were helical as expected, if each construct forms a coiled coil (Figure 2, C and D). However, the majority of the mutant constructs were less helical than the WT constructs, as measured by mean residue ellipticity (MRE) at 222 nm (Figure 2, A and B). Helicity was significantly decreased for all the mutations, with the exception of MYH7 E1610K (Figure 2C). Thus, either mutation to proline or deletion does disrupt the secondary structure of the coiled coil.

We then assessed the thermal stability of each of the mutations by evaluating the change in the MRE at 222 nm (Figure 2, D and E) from 10°C to 80°C and by calculating the temperature at which half the protein melted (T_m_). Four of the 9 MYH7 mutations tested here showed changes in thermal stability. L1492P, which had the largest decrease in helicity (Figure 2C), also had the largest decrease in thermal stability (Figure 2F). K1729del, which had strongly reduced helical content, had a slightly lower thermal stability, but the decrease was not significant. In contrast, E1610K, which had a similar helical content to WT (Figure 2C), had an increased thermal stability (Figure 2F). The remaining MYH7 mutations showed no clear correlation between helical content and thermal stability. The helical content for the MYH2 mutation L1870P was significantly reduced (Figure 2C). While its T_m_ was reduced (Figure 2F), this difference was not significant.

**Figure 3:**
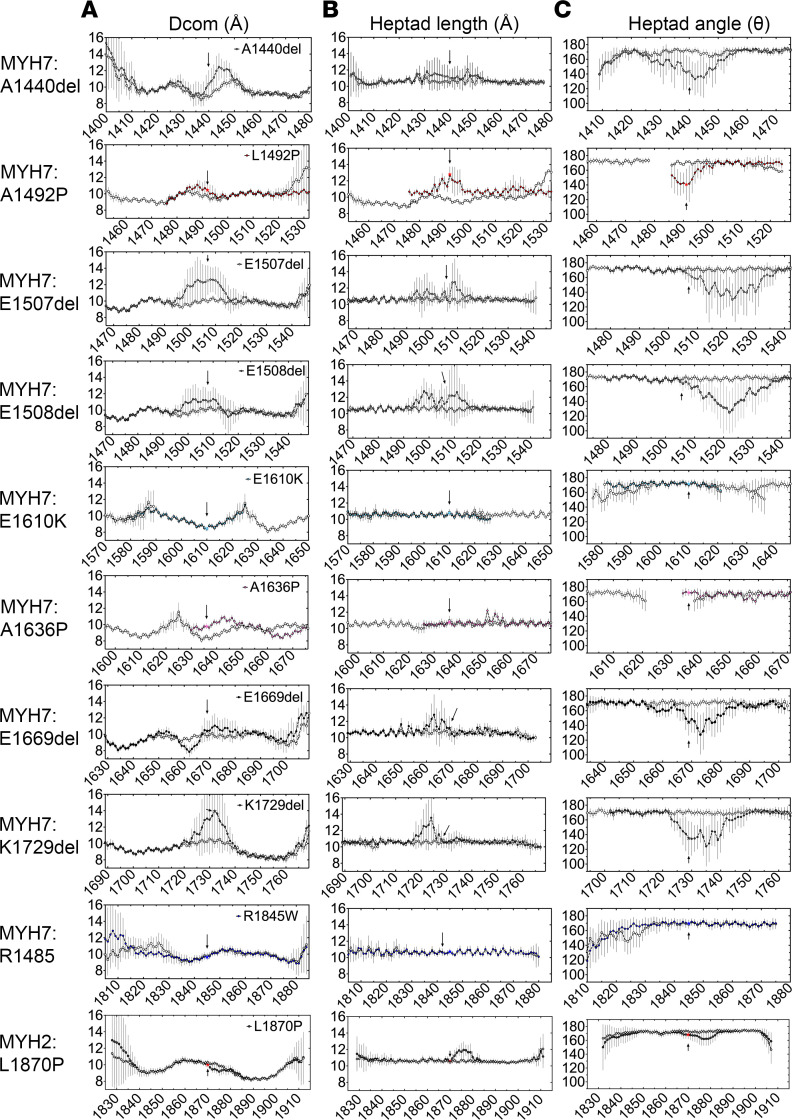
Molecular dynamics simulations of mutant coiled coil models. (**A**) The distance between the helices (D_com_) for MYH7 and MYH2 mutants as a function of residue position compared with WT (open circles). (**B**) Heptad length, the distance between Cα atoms in residues at positions i and i + 7, averaged across both chains of the coiled coil. (**C**) Interheptad angle, the angle between the lines linking Cα atoms in residues at positions (i – 7), (i), and (i, i + 7), averaged across both chains of the coiled coil. Filled circles, results for the mutant; open circles, results for the WT. Arrows and star indicate the position of the mutated residue in each of the plots. Three independent simulations were performed. Data are shown as mean ± SD for each data point between simulations.

To better characterize local perturbations to the structure of the coiled coil that could arise from each of the mutations, we performed molecular dynamics simulations similar to those we did previously (21). This allowed us to determine the local distances between the 2 helices (D_com_), the local heptad length, and the angle between adjacent heptad sections in each helix (Figure 3). In this approach, shorter WT constructs were used either based on existing crystal structures or de novo models created using BEAMMOTIFCC (22).

All the deletion mutants had a strong effect on D_com_, showing clear evidence for separation of both helices. In the deletion mutants in MYH7, the 2 helical strands tended to separate before and after the mutation. For E1669del, the 2 helical strands separated in opposite ways before and after the mutation (Figure 3A). Each of these mutations showed effects of local heptad length close to the site of the mutation (Figure 3B) and a pronounced effect on heptad angle (Figure 3C). Thus, each of the deletion mutants considerably disrupted the structure of the coiled coil close to the mutation, introducing large local kinks in the coiled coil tail in order to accommodate the break in the heptad repeat and bring the coil back into register. This is consistent with the reduced helicity for each of these mutants in LMM, measured experimentally.

For the MYH7 proline mutation, L1492P, the strand separation around the site of the mutation was relatively small, and there was a more pronounced increase in heptad length and a decrease in heptad angle (Figure 3, A–C). For A1636P, there was an increase in strand separation around the site of the mutation, but there was little effect on heptad length or angle. These differences may help explain the larger reduction in helicity and thermal stability experimentally observed for A1492P compared with A1636P. For E1610K and R1845W, there were little strand separation around the site of the mutation (Figure 3A), little change in heptad length (Figure 3B), and little change in heptad angle (Figure 3C), consistent with small or no effects on helicity for these 2 mutants.

Finally, the MYH2 proline mutation, L1870P, showed a small increase in strand separation, a more pronounced increase in heptad length, and a decrease in heptad angle (Figure 3, A–C), somewhat similar to that observed for L1492P. This is consistent with the decreased helicity and thermal stability measured for this mutant.

### LMM mutations affect GST-LMM filament formation in vitro, but GFP-MHC constructs can incorporate into sarcomeres in vivo.

**Figure 4:**
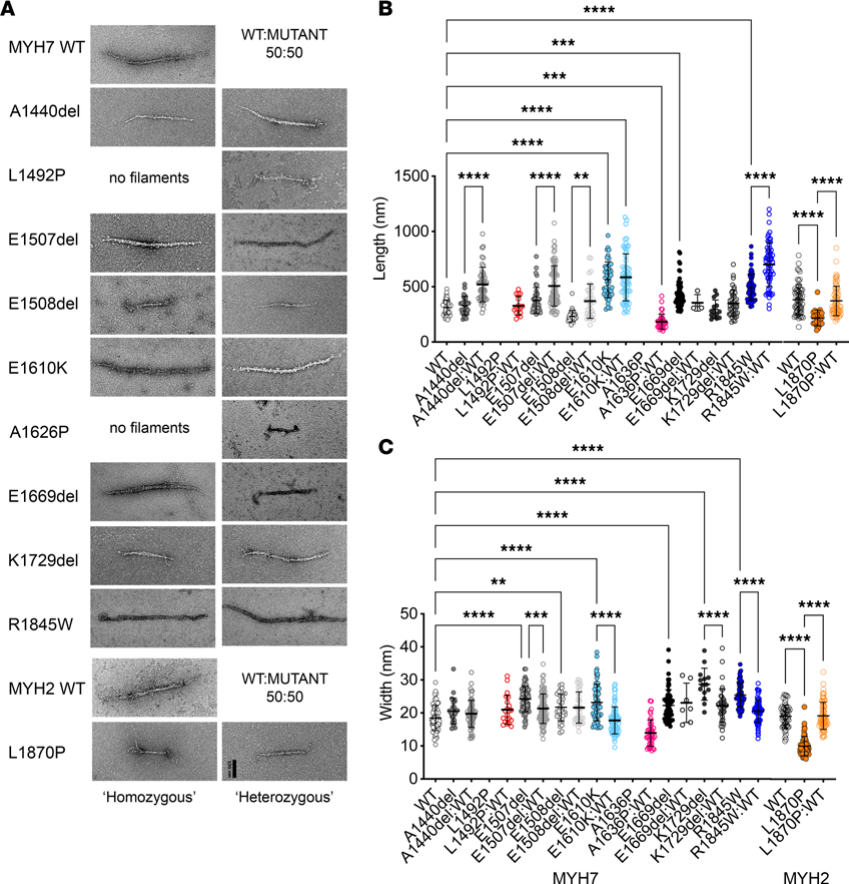
Effects of mutations on aggregation of GST-LMM into minifilaments in vitro. (**A**) Examples for GST-filaments, negatively stained and imaged using electron microscopy. The left column shows GST-LMM filaments that are effectively homozygous, as the filaments were generated from a pure population of each type of construct. No images are shown for either of the *MYH7* mutations to proline (L1492P or A1630P), as filaments did not form under these conditions. The right column shows heterozygous GST-LMM filaments, in which the mutant LMM was mixed 50:50 with WT LMM. Scale bar: 100 nm. (**B**) Filament lengths for homozygous populations and the widths. (**C**) Filament lengths for heterozygous populations and the widths. The mean ± SD is shown for each set of measurements. Significant differences are as shown. The 1-way ANOVA with Dunnett’s test post hoc correction was used with **P < 0.01; ***P < 0.001; ****P < 0.0001.

GST-LMM molecules form short filaments in vitro in low–ionic strength conditions, enabling us to characterize the effects of mutations on filament formation (21). LMM without GST forms extensive paracrystals that are more difficult to analyze (23). We might expect that small changes in secondary structure of LMM might affect its ability to aggregate into filaments, since this depends on precise interactions between neighboring molecules that could be locally disrupted as a result of the mutations. To assess this, filaments were formed by mixing either homogenous WT or mutant GST-LMM molecules (homozygous conditions) or by mixing 50:50 WT/mutant LMM molecules (heterozygous conditions) and then imaging the filaments using negative stain EM (Figure 4A). The lengths and widths of these filaments were then measured. Filaments formed under these conditions are not the same as filaments in vitro, in which there are also accessory proteins such as titin and MyBPC, but they do reveal effects of mutants on the aggregation of LMM into minifilaments.

Of the 9 MYH7 mutations investigated for homogeneous (homozygous) mixtures of GST-LMM, neither of the proline mutants (L1942P and A1626P) formed minifilaments in vitro (Figure 4A). When 50:50 mixtures of WT and mutant GST-LMM constructs were mixed (heterozygous), filaments for the 2 MYH7 proline mutants could now be observed (Figure 4A). L1492P/WT filaments had similar dimensions to WT filaments, while A1636P/WT filaments were shorter and narrower (Figure 4, B and C). The MYH2 proline mutant (L1870P) did form filaments from homogeneous mixtures, which were shorter and narrower than WT (Figure 4, B and C). When mixed 50:50 with WT, filament dimensions were similar to WT.

The effects of the deletion mutants in MYH7 of GST-LMM filament formation were variable (Figure 4, B and C). In homogeneous mixtures, A1440del assembled normally, but in a 50:50 mixture with WT, the filaments were significantly longer. E1507del homogeneous filaments were significantly longer and wider than WT, and the same trend was observed in 50:50 mixtures with WT. E1508del homogeneous filaments were significantly shorter and wider than WT, and in 50:50 mixtures, length returned to normal, while width remain increased. E1669del homogeneous filaments were significantly longer and wider than WT, and in 50:50 mixtures with WT, length returned to normal while width remained increased. Finally, K1729del homogeneous filaments were significantly wider than WT filaments, as were 50:50 mixtures with WT.

A significant increase in GST-LMM filament length and width was observed for the 2 missense mutants, E1610K and R1845W, for both homogenous and 50:50 WT/mutant mixtures (Figure 4, B and C). Overall, the majority of the mutants tested here have effects on GST-LMM aggregation into minifilaments, suggesting that the mutations disrupt aggregation into filaments in vitro and these effects are mostly not rescued by mixing in WT GST-LMM.

**Figure 5:**
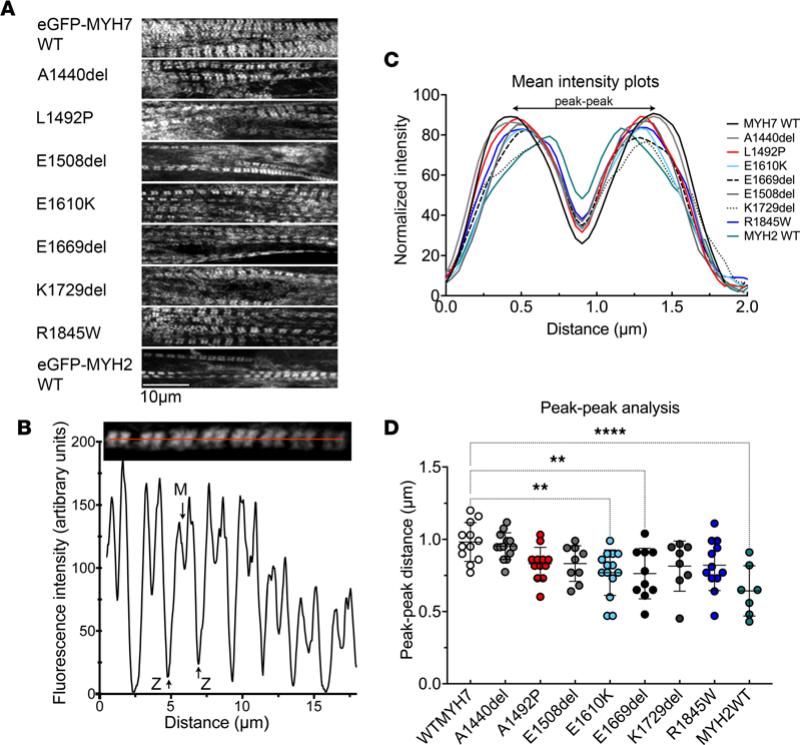
Analysis of incorporation of mutant isoforms of eGFP–MHC into muscle sarcomeres in cultured skeletal muscle myotubes formed by C2C12 cells. (**A**) Images of eGFP-MHC^+^ myofibrils within cultured skeletal muscle myotubes for WT MYH7 and each of the mutants. (**B**) An example image of a single eGFP-MYH7^+^ myofibril together with its associated line profile. Positions of the minimum intensity value (at the M-line) and ends of the sarcomere (at the Z-disc) are indicated. (**C**). Mean fluorescence intensity profiles for eGFP-MHC organization across a single sarcomere (from Z-disc to Z-disc) for WT and each mutant. SD bars are omitted for clarity. The mean plot profile was calculated from a minimum of 7 myofibrils taken from different myotubes from 3 biological replicates (mean, SD, and *n* values are in Supporting Data Values). The MYH2 L1871P mutant did not express well enough to quantify sarcomere incorporation. (**D**) The peak-to-peak distance across the M-line for each of the GFP-MHC constructs, together with the mean ± SD values. Significant differences were calculated using a 1-way ANOVA with Dunnett’s test post hoc correction. **P < 0.01 and ****P < 0.0001.

Finally, we determined the effects of these *MYH7* and *MYH2* mutations on sarcomere incorporation by expressing eGFP-full length MHC constructs in differentiated myotubes. In this case, the myosin can coassemble with endogenous myosin, as well as thick filament accessory proteins, to form filaments. Despite effects on filament formation in vitro, all of the mutations we tested were able to incorporate into nascent muscle sarcomeres (Figure 5A). The eGFP is fused to the N-terminus of the MHC, just upstream from the motor domain, which results in 2 fluorescent bands per sarcomere, with low GFP signal in the central M-band region (Figure 5B) and in the Z-disc regions at the end of each sarcomere. The low signal in the M-band arises from the lack of myosin heads in this region due to the antiparallel packing of the myosin tails in the center of the thick filament.

An analysis of the intensity profiles for eGFP-MHC (Figure 5B) across individual sarcomeres (Figure 5C) showed that the overall incorporation is similar for all of the constructs. In each of these sarcomeres, 2 highly fluorescent bands either side of the M-band, where fluorescence intensity is reduced, could be observed (Figure 5C). In most cases, the distance between the peak fluorescence in each of these 2 bands is similar (Figure 5, C and D), with the exception of E1610K and E1669del, perhaps indicating a small effect on the ability of these 2 mutants to incorporate uniformly across the muscle sarcomere. However, the WT GFP-MYH2 also shows a reduction in peak-to-peak distance (Figure 5D) as well as an apparently smaller reduction in fluorescence at the M-band (Figure 5C). Qualitatively, compared with WT MYH7 GFP, sarcomeres appear less extensive for all of the mutants and for MYH2 WT. This may indicate that these GFP-MHC constructs are slower to incorporate and form ordered sarcomeres than WT MYH7. However, overall small disruptions to structure as observed in in vitro experiments appear to be accommodated in the cellular environment.

### In the presence of human LMM mutations, myosin heads are disordered.

To test whether the myosin mutations have an impact on myosin filament length, myosin head order, and myofilament organization in vivo, we tested the same mutations in human tissue samples (Supplemental Table 1; supplemental material available online with this article; https://doi.org/10.1172/jci.insight.172322DS1). In this case, the mutants are heterozygous. Of the 255 muscle fibers (at least 10 myofibers for each individual) analyzed, 161 expressed the β/slow MHC isoform encoded by *MYH7,* with the remainder expressing the type IIA MHC isoform encoded by *MYH2*. Immunofluorescence staining of both patient and control myofibers expressing either β/slow or type IIA MHC showed that muscle sarcomeres were well preserved, as indicated by regular striated arrays of myosin filaments (Supplemental Figure 1A). The length of these filaments (Supplemental Figure 1, B and C) was consistent with the interindividual and intermuscle heterogeneity reported previously (24).

Equatorial reflections related to myosin and actin filaments (1,0 and 1,1, respectively) as well as high-quality meridional reflections related to myosin repeats (M3 and M6), were obtained by x-ray diffraction of control and patient muscle bundles in relaxing conditions. Other meridional or layer line reflections were not analyzed, since they were difficult to distinguish in the patients. To ensure reliable results and avoid misinterpretation, we pooled all the patients’ data together and compared these with images acquired for controls. This revealed that the intensity ratio (1,1 to 1,0 ratio) was greater in the patients than in the controls, indicating a shift of myosin heads toward the actin filaments. Additionally, the intensity of the M3 layer line, representative of the distance between myosin crowns (typically 14.3 nm), was lower in the muscle fiber bundles from patients than in controls, suggesting that the heads are more disordered in samples from patients. The intensity of the M6 layer line, which is thought to be related to the helical order of the heads on the thick filament, indicative of myosin filament backbone periodicity/compliance, was preserved (Supplemental Figure 2). The spacings of the M3 and M6 meridional layer lines were unchanged between controls and patients (Supplemental Figure 2). Altogether, these indicate a specific myosin disorder with heads leaning toward the thin filament.

### In the presence of human LMM mutations, the myosin DRX/SRX ratio is increased.

To estimate the ratio of myosin heads in the DRX compared with those in the SRX state, we used Mant-ATP chase experiments (8, 13). This approach relies on a fluorescent ATP named Mant-ATP and on the fact that the rate of ATP consumption by DRX heads is approximately 5- to 10-fold higher than that by SRX heads. Typically, when Mant-ATP is used, the decay of fluorescence intensity over time is best fitted by a double exponential curve, allowing the separation of fast and slow phases (indicative of DRX and SRX states, respectively).

**Figure 6:**
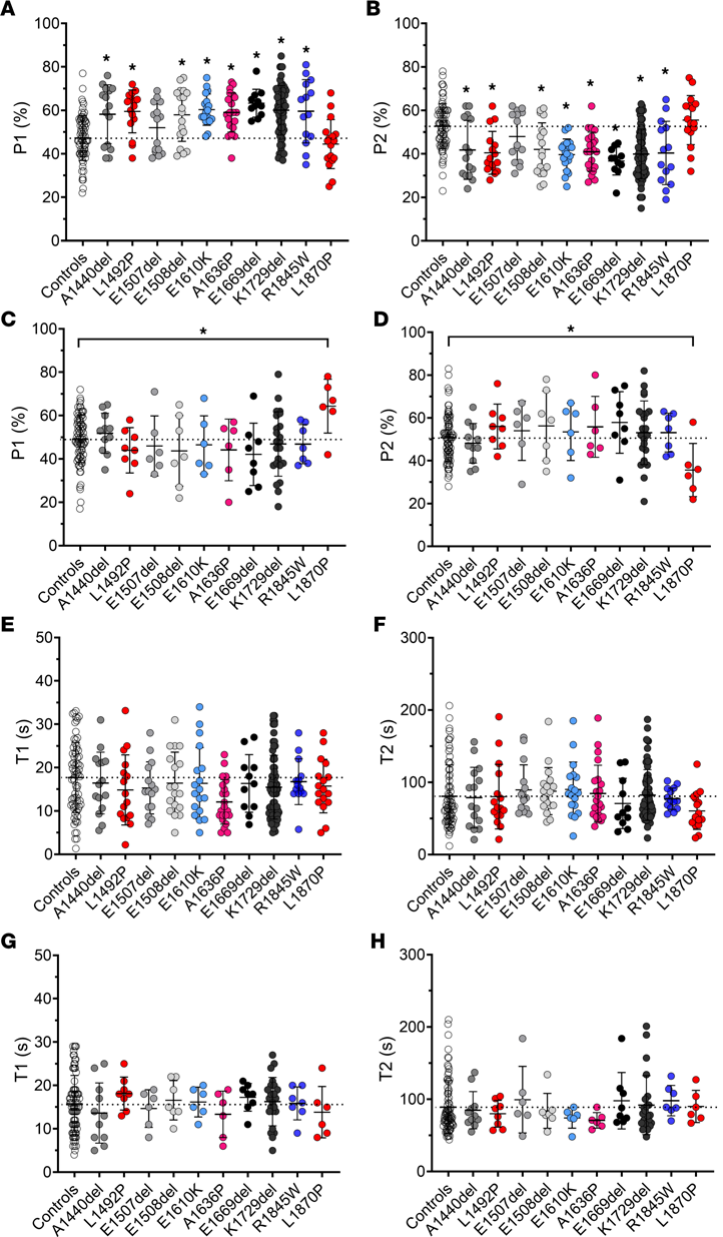
Mant-ATP chase experiments to estimate DRX/SRX ratios. Muscle fibers were isolated from controls (CTL) or from patients with *MYH7* (MYH7) or with *MYH2* (MYH2) mutations. The proportion of myosin molecules in the disordered-relaxed (P1) and super-relaxed states (P2), as well as their respective ATP turnover lifetimes (T1 and T2), are presented. Data are separated according to the myosin heavy chain expression of individual fibers: either β/slow (graphs **A**, **B**, **E**, and **F**) or type IIA (graphs **C**, **D**, **G**, and **H**). For the β/slow isoform, *n* = 73 were from CTL and *n* = 214 were from patients. For the type IIA isoform, *n* = 81 were from CTL and *n* = 88 were from patients. Mean ± SD also appear on the graphs. Significance indicates a difference with control. The 1-way ANOVA with Dunnett’s test post hoc correction was used with *P < 0.05. Typical Mant-ATP chase experimental data showing exponential decays are presented in Supplemental Figure 4.

A total of 456 muscle fibers were tested (more than 20 myofibers for each of the 7 controls and for each of the 23 patients; see list in Supplemental Table 1). Out of these 456 myofibers, 287 expressed the β/slow MHC isoform. P1, indicative of DRX, was increased (P2, indicative of SRX, was decreased) in myofibers expressing β/slow MHC obtained from patients with *MYH7* mutations, compared with controls or with patient fibers with *MYH2* mutations (Figure 6, A and B). Similarly, P1 was increased (P2 was decreased) in myofibers expressing type IIA MHC from patients with *MYH2* mutations compared with controls or patients with *MYH7* mutations (Figure 6, C and D). The ATP turnover lifetime of individual myosin molecules in DRX and SRX states (as assessed by T1 and T2, respectively) was, however, not affected in any of the conditions (Figure 6, E–H). Overall, this suggests that, in the presence of heterozygous *MYH7* and *MYH2* mutations, myosin heads are not sequestered properly onto the filament backbone, potentially increasing the number of myosin molecules in an ON state ready for crossbridge recruitment (25).

### Modeling suggests that a greater number of myosin motors in the DRX state leads to a small increase in contractility.

**Figure 7:**
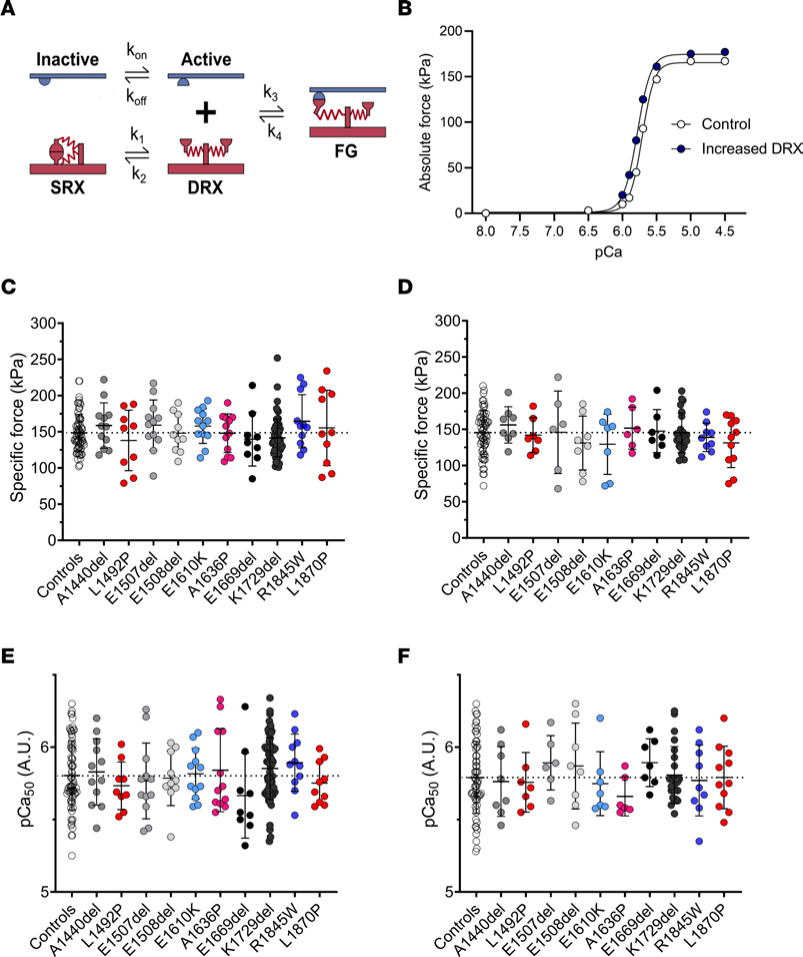
Modeling and experimental cellular force production. (**A**) The 3-component model used for our simulation from ref. 26 with SRX, DRX, and FG (force-generating) states. (**B**) The result of the simulations. Experimental specific force and Ca^2+^ sensitivity for muscle fibers isolated from controls (CTL) or from patients with *MYH7* (MYH7) or with *MYH2* (MYH2) mutations. Data are separated according to the myosin heavy chain expression of individual fibers: either β/slow (graphs **C** and **E**) or type IIA (graphs **D** and **F**). For the β/slow isoform, *n* = 72 were from CTL and *n* = 161 were from patients. For the type IIA isoform, *n* = 64 were from CTL and *n* = 92 were from patients. Mean ± SD appear on the graphs. The 1-way ANOVA with Dunnett’s test post hoc correction was used, but no significant differences were seen.

To predict whether the increased DRX/SRX ratio could solely affect the number of myosin heads binding to actin and the cellular force production, we applied (in the absence of any LMM mutation) a spatially explicit model of myofilament-level contraction that simulates contractile properties generated by a computational tool, FiberSim 2.1.0 (https://campbell-muscle-lab.github.io/FiberSim/) (26). An increase in myosin heads in the DRX state led to a small increase in isometric force (at maximal and submaximal activation levels) and increased Ca^2+^ sensitivity (pCa_50_ = 5.77 compared with pCa_50_ = 5.69) (Figure 7, A and B).

To complement these computational findings, we measured the contractility of human individual membrane-permeabilized myofibers. A total of 389 myofibers were evaluated (more than 10 myofibers for each of the subjects). Out of these 389 myofibers, 233 were positive for the β/slow MHC isoform. However, there were no significant differences in the isometric force produced (Figure 7, C and D) or Ca^2+^ sensitivity (Figure 7, E and F) between myofibers from patients and controls. This is likely due to the cell-to-cell variability.

## Discussion

Our findings support the concept that mutations in *MYH7* and *MYH2* that lead to myosinopathy could increase the number of DRX heads in relaxed muscle. We found that the majority of the mutations affected secondary structure of the myosin coiled coil and filament formation in vitro. Even though the mutant isoforms appeared to incorporate normally into individual sarcomeres in vivo, small effects of the myosin coiled coil structure have the ability to disrupt packing in the filament. These could induce the destabilization of shutdown myosin heads, increasing the DRX/SRX ratio, and thus increasing ATP usage in relaxed muscle fibers. Although not tested here, this could also arise through disruption of LMM packing into the filament through its interactions with accessory proteins, such as titin and myosin recently reported in 2 recent CryoEM structures of the C-zone of the thick filament (4, 5). Besides these, we did not observe any substantial effect on the muscle fiber force generating capacity. Overall, our results provide important insights into the pathogenesis of skeletal myosinopathies and raise the idea that these particular genetic diseases are underappreciated and lead to an unexpected increase in ATP usage rather than contractile impairment.

### Increased DRX/SRX ratio as a major pathophysiological mechanism in the presence of LMM mutations.

Our observation of altered DRX/SRX ratio has previously been shown to be involved in the pathogenesis of HCM arising from *MYH7* mutations. In the latter case, these have been linked to a large number of subtle residue replacements in the myosin motor domains in a region termed the mesa. Mutations in residues in this region interact with the first part of the coiled coil and can directly interfere with the formation of the IHM essential for myosin head sequestration, promoting the cardiac phenotype (27–29), and this has been further explored in a recent high-resolution structure of shutdown cardiac myosin (30). Here, mutations in the LMM region would need to have a more indirect effect on the stabilization of IHM heads, through a more general destabilization of filament packing. An increase in DRX heads, and thus an increase in ATP usage, is consistent with clinical findings that demonstrate changes to muscle bioenergetic and metabolic profiles in patients. Specifically, ultrastructural and histological analyses have shown the accumulation, proliferation, and abnormal structure of subsarcolemmal mitochondria (31, 32).

Although more comprehensive studies directly linking myosin metabolic power with mitochondria or whole-body metabolism are needed, an excessive myosin ATP consumption could trigger a metabolic switch away from glycolytic pathways toward a greater reliance on mitochondrial oxidative phosphorylation to meet the increased energy demand. The human resting skeletal muscle metabolic rate is low but accounts for 25% of the obligatory whole-body thermogenesis (33). An increased DRX/SRX ratio by 10%–20% (as found here for the patients with mutations in *MYH7* and *MYH2*) would double skeletal muscle thermogenesis, increasing thermogenesis and the whole-body basal metabolic rate by approximately 16% (8, 13). Regardless of inactivity or limited physical exercise, over a period of a year, the 20% increase in myosin DRX would provoke a mean weight loss of about 7 kilograms (13). Altogether, this would support the amount of clinical observations reporting that patients diagnosed with skeletal myosinopathies are lean or underweight, despite being inactive and sedentary. Besides a metabolic imbalance, how the higher proportion of myosin heads in the DRX state is linked to other muscle symptoms (such as weakness) in patients with skeletal myosinopathies remains unclear. Our modeling and experimental findings of preserved cellular contractile generating capacity in the presence of enhanced DRX/SRX ratio strongly indicate that other unknown and unrelated mechanisms may occur. These would need to be explored in future studies.

### Dysregulated coiled coil structure and filament packing as a potential cause of the increased DRX/SRX ratio.

In contrast to *MYH7*-driven HCM (27–29), here, the reduced myosin head sequestration in patients cannot be attributed to a direct effect of the mutations on the IHM through the myosin mesa but rather to other processes involving the LMM region. LMM is highly conserved among vertebrates, emphasizing the importance of its amino acid arrangement for the formation of the heptad repeats (7 residue repeat pattern *a*–*g*) within the coiled coil and for myosin filament packing (34). Interestingly, here, most of the subtle *MYH7* and *MYH2* mutations affect residues in *b*, *c*, and *f* positions of the charged exterior portion of the coiled coil (34). Of the mutations we have studied here, the deletion mutations in E1507, E1508, E1669, and K1729 occur in regions of LMM that are strongly negatively charged (Supplemental Figure 3). Deletion of these residues would be expected to decrease the overall negative charge of this region in addition to disruption of the coiled coil structure, and both of these factors could contribute to altered filament packing in thick filaments. E1610 and R1845 are both in regions that are of strong positive charge and likewise have the potential to disrupt myosin packing into thick filaments through this change to the overall positive charge.

While it seems likely that the main effect of destabilizing packing of LMM into filaments is to destabilize formation of the IHM in the SRX state, it is worth noting that the filaments themselves may be destabilized and that rates of incorporation of the mutant myosins may differ from that of WT. As nascent myofibrils form, they gradually align laterally, resulting in the cross-striations observed in adult skeletal muscle (35). While we observed evidence for myofibrillar alignment for WT GFP-MYH7, this was less evident for the mutant MYH7 constructs. One potential explanation for this is that the rate of incorporation of the mutant MYH7 constructs is reduced; thus, myofibril formation is less extensive. We previously found that a specific MYH7 mutation that leads to HCM (N1327K) that reduced the helicity of the coiled coil in vitro also reduced its incorporation into sarcomeres in isolated adult rat cardiomyocytes and increased the rate of its exchange into thick filaments (36), suggesting that this myosin is less stably incorporated into filaments. However, this mutation did not affect force generation. Moreover, our IF and x-ray diffraction (XRD) experiments of human fibers harboring the mutations studied here did not show any abnormality that would reveal incorporation issues or the presence of aggregates.

In striated muscle myosins, the IHM is thought to mainly depend on the interaction of the 2 heads with each other and with the first part of the coiled coil (S2). However, the proportion of myosin heads in the SRX state appears to strongly depend on the molecular state of the myosin, being low in single molecules and much higher in molecules organized into filaments (reviewed in ref. 27), where myosin molecules can interact with tails in the filament backbone as well as with thick filament proteins such as MyBPC and titin. This raises the possibility that interactions with LMM may also play a role in stabilizing the SRX state in the filament. In isolated, shutdown, smooth muscle myosin, the distal coiled coil region (LMM) interacts with the so-called blocked head of the IHM, with key interactions between LMM and the SH3-like fold at the N-terminus of the motor domain as well as with the converter domain (37, 38). A similar interaction between LMM and the IHMs in cardiac and skeletal muscle filaments could help to keep the myosin heads in the IHM state, by inhibiting the movement of the converter domain of the blocked head (27). Our results indicate that, while the mutations likely affect the local structure of the coiled coil, they do not have strong effects on sarcomeric incorporation in vivo. However, they could affect the ability of the myosin to adopt an SRX state through a subtle disruption of myosin filament packing.

### Conclusion.

Taken together, our findings demonstrate a clear pathogenic effect of *MYH7* and *MYH2* mutations (associated with congenital myopathies) on myosin coiled coil structures. Additionally, our results highlight that, in resting human myofibers from patients, the myosin-stabilizing conformational state is altered, increasing the DRX/SRX ratio and basal ATP demand. All of these changes do not substantially affect the cellular force–producing capacity. Nevertheless, they give valuable insights into skeletal myosinopathies, the unexplained odd appearance of energetic proteins (e.g., mitochondria), and the potential benefits of myosin-linked drugs targeting its ATPase activity.

## Methods

### Generation of LMM mutant constructs.

The cDNA used in these experiments encodes sequences from the human *MYH7* (P12883, Uniprot) and MYH2 (Q9UKX2, Uniprot). The cDNA for *MYH7* and the *MYH7* LMM (residues 1280–1936) constructs were generated as described previously (21). The LMM coding region was subcloned into pGEX-6P-1 (GE Lifesciences) as previously described (21), and this adds a GST sequence and a PreScission protease site at the N-terminus. The desired mutations for *MYH7* were introduced using the Quick ChangeXL II Site Directed Mutagenesis Kit (Agilent) according to the manufacturers’ instructions. Each construct was sequenced to verify that the desired mutations had been introduced and to confirm that no other mutations had been introduced. The cDNA for full-length *MYH2*, together with a mutated form (L1870P), was obtained from GenScript. The cDNA for *MYH2* LMM region (residues 1223–1941) was subcloned into the pGEX-6P-1 (GE Lifesciences) for expression and purification. All LMM constructs were designed to start and finish on an amino acid in the d position of the heptad repeat.

### Expression and purification of LMM.

The WT and mutant constructs were transformed into *E*. *coli* Rosetta 2 (Novagen); a single colony was inoculated into a 5 mL starter culture and grown overnight before being added to 500 mL Auto-Induction media (formulation [g/L]) — 12 g tryptone, 24 g yeast extract, 0.15 g MgSO_4_, 3.3 g (NH4)_2_SO_4_, 6.5 g KH_2_PO_4_, 7.1 g Na_2_PO_4_, 0.5 g glucose, 2 g alpha lactose (pH 7.0), and grown at 20°C for 18 hours. Cells were harvested and the pellets stored at −80°C. For protein purification, the pellets were resuspended in lysis buffer (PBS, 350 mM NaCl, 1 mM DTT, 1 mM EDTA, 200 μg/mL lysozyme, 0.1% Triton X-100, protease cocktail inhibitor tablet [Roche; pH 7.5]) for 30 minutes at room temperature on a roller. The lysates were sonicated on ice (6 cycles of 10 seconds on, 10 seconds off), and the cell debris was pelleted by centrifugation (30,000*g*, 30 minutes, 4°C). Proteins were purified by GST-tag affinity chromatography using Glutathione Sepharose 4B (GE Lifesciences). First, cleared lysates were added to the equilibrated 1.5–2 mL glutathione Sepharose 4B and washed with 100 mL of wash buffer (PBS, 350 mM NaCl, 1 mM DTT, 1 mM EDTA [pH 7.5]). For CD experiments, the LMM was liberated from the column matrix by overnight incubation with PreScission Protease in cleavage buffer (50 mM Tris–HCl, 150 mM NaCl, 1 mM EDTA, 1 mM DTT [pH 7.5]) at 4°C. The constructs were then concentrated with a vivaspin 6 and dialyzed against CD buffer (500 mM NaCl, 10 mM phosphate buffer [pH 7.5], 1 mM DTT). For EM studies, the GST tag was not cleaved away from LMM constructs, and the intact GST-LMM was eluted from the column by using 20 mM reduced glutathione. To assess the purity of the protein, fractions were collected, the protein content and purity were assessed by SDS-PAGE, and protein concentration was measured using the BCA Assay (MilliporeSigma).

### CD measurements.

CD spectra for LMM constructs from which GST had been removed were measured at a temperature of 10°C at wavelengths from 260 to 190 nm using an APP Chirascan CD spectropolarimeter. The CD buffer contained 500 mM NaCl, 1 mM DTT, and 10 mM phosphate buffer (pH 7.5). The concentrations for all LMM constructs were 150–250 μg/mL. Scans at 10°C were repeated twice, and, at minimum, 3 experiments were performed. To calculate any significant difference, we compared the 222 nm MRE values for all the mutant constructs with that for WT using a 1-way ANOVA, together with a post hoc Dunnett’s test. A fully (100%) helical construct was considered to have an MRE value at 222 nm of 36,000 (100%) (39). This value was used to estimate the percentage of helical content of the constructs. Thermal melting measurements were taken at 1°C increments from 10°C to 80°C, with 1°C/min heating rate, in the same buffer.

### Electron microscopy (EM).

GST-LMM filaments were generated by rapidly diluting GST-LMM into a low-salt solution (150 mM NaCl, 10 mM MOPS [pH 7.2], 1 mM EDTA). The mixture was then left on ice for 2 minutes to allow for filament formation. The final concentration of GST-LMM was 0.3–0.5 μM. In total, 5 μL was then loaded onto a carbon-coated copper grid, previously irradiated using a PELCO easiGlow Glow Discharge Cleaning system (TedPella). The sample was allowed to adhere for 10 seconds before the sample was flicked away and stained with 1% uranyl acetate. The grids were imaged using an FEI Tecnai TF20 microscope at 120 kV, and micrographs were recorded using a 4k × 4k Gatan CCD camera (pixel size of 0.351 nm). Measurements of filament lengths were performed using ImageJ (FiJi) software (40). Graphs and analyses were generated using Prism (GraphPad) software.

### Molecular dynamic simulations.

Simulations were performed as previously described (21). Simulations using explicit solvent were performed using the CHARMM-36 force field parameters (41) with TIP3P water on fragments that were 13 heptads (91 residues) long. A starting structure for the composite model of residues 1526–1689 within the LMM region of human β-MHC (42) was generously supplied by Ivan Rayment (University of Wisconsin–Madison, Madison, Wisconsin, USA) and Qiang Cui (Boston University, Boston, Massachusetts, USA). MD simulations were also performed on shorter segments of this same part of the β-MHC coiled coil for which experimental atomic structures are available (43). For these, the N-terminal globular Xrcc4 moieties were removed using Chimera (44). Specifically, residues 1562–1608 were taken from 5CJ4 (chain A, residues 1562–1615; chain B, residues 1562–1608; ref. 43). Residues 1590–1657 were taken from 5CHX (43), and residues 1631–1689 were taken from 5CJ0 (42). Methylated lysine side chains (modified to facilitate crystallization in 5CJ4 and 5CHX) were converted back to unmodified lysine. In all cases, N-termini were capped with acetyl groups, and C-termini were capped with N-methylamide for both chains. Atomic structures equivalent to the WT composite models were also made for all mutants. To generate the substitution mutants, the most probable rotamer for each chain was substituted using the Structure Edit function within Chimera (44).

For skeletal myosin, the coiled coil tail model (7KOG) from the *Lethocerus indicus* thick-filament cryo-EM reconstruction (45) was used as a starting model. The skeletal myosin sequence was threaded onto the 7KOG model using Modeller 9.18 (46). A 13-heptad segment (residues 1825–1915) was extracted to yield a WT model. The L1870P mutant was generated by mutating L1870 to a proline using the Structure Edit function within Chimera as before.

Generation of a starting structure for the deletion mutants was performed as described previously (21). Producing a model for deletion mutants was more challenging than for the substitution mutants, since simply deleting a residue from the WT model and rebonding the chains across the gap produces a break in the coiled coil structure that would not occur during formation of the mutant coiled coil in vivo. We therefore built a noncanonical coiled coil ab initio for each deletion mutant, along with a corresponding WT equivalent model using the program BEAMMOTIFCC (22). These models used noncanonical structures for the skip residue (where applicable) and for the deletion site. Briefly, the initial WT model was built using the following values: 3.617 residues per turn of helix, an axial translation per residue of 1.495 Å, and a relative rotation of the 2 helical strands of 210°. The major helical radius used was 4.9 Å, which was the average major helical radius calculated along the coiled coil from simulations of the composite PDB model. The smoothing parameter b was varied to find a value that resulted in simulation average properties that best matched those from simulating the composite PDB model (b = 0.03 was the optimum). The structure is defined in the program by a pattern of “motifs” that link equivalent residues in the sequence; the motif for a canonical coiled coil is 7 amino acid residues. To accommodate the skip residues E1582 and G1807, a 29-residue motif replaced four 7-residue motifs between F1565 and V1594 and between L1796 and E1825, respectively. We validated the method by comparing these 91-residue WT models with the composite model, where applicable.

Starting simulations from our ab initio model gave rise to average properties that all matched very well with those found for simulations initiated from the composite model, showing that the method copes well with skip residues. We then used the same approach to build deletion coiled coil mutants with a smooth all-helix structure from which to initiate simulations. To build a noncanonical deletion model with a continuous helical structure, in addition to the skip motif modification (where applicable), a 27-residue motif replaced 4 canonical 7-residues motifs. The BEAMMOTIFCC program was modified in order to implement the 27-residue motif, which is defined to contain 8 helical turns (like in the WT four 7-residue motifs that it replaced) to ensure a left-handed coiled coil (refer to the *N* value in ref. 22 for details). BEAMMOTIFCC provides a backbone structure for the coiled coil model. Side-chain atoms were added to the model using SCWRL4 (47). N-termini were again capped with acetyl groups, and C-termini were capped with N-methylamide. Simulations were run as for the composite model with an additional minimization and equilibration run with restrained backbone atoms to allow for side-chain–only equilibration (10,000-step minimization, 0–300 K heating protocol, and 100,000-step preequilibration performed using AMBER; https://ambermd.org/index.php) prior to all atom minimization.

CHARMM-GUI (48) was used to add a 1.5 nm surround of water molecules, and Na^+^ and Cl^−^ ions were then added to neutralize the construct and give a NaCl concentration of ~150 mM. A 10,000-step minimization, 0–300 K heating protocol, and short preequilibration run (100,000 steps) were performed using AMBER. Data are taken from simulation runs lasting at least 1 μs across at least 3 separate runs initiated using a random number seed in AMBER (49) at 300 K. The time step used was 4 fs, and trajectory frames were recorded every 25,000 steps. The first 10 ns of each run was removed prior to analysis to avoid starting structure bias. Simulation trajectories were analyzed using CPPTRAJ (50). The helicity of both chains was calculated using backbone dihedral angles and the method previously described (41). The mean ± SD D_com_ values were calculated using a moving window to include the positions of 7 Cα (3 N-terminal and 3 C-terminal to the marked residue in each chain). Similarly, average heptad lengths along each helix were calculated using a moving window average of Cα positions in 7 (i) residues and their (i + 7) residue partners in the sequence. The interheptad angles were measured between Cα atoms in residues at sequence positions (i – 7), i, and (i + 7).

### Expression of eGFP–β-MHC in cardiomyocytes and myotubes.

C2C12 myoblasts, purchased from Public Health England culture collections, were used to generate skeletal muscle myotubes in culture. They were maintained in DMEM, with high glucose and Glutamax, supplemented with 20% FCS and 1% penicillin and streptomycin (diluted from stocks containing 10,000 U/mL) and used at low passage (<10 passages). The cells were differentiated in DMEM supplemented with 2% horse serum and 1% penicillin and streptomycin. The adenovirus expression vector, pDC315, was used to express the full-length heavy chain of MYH7 (P12883, Uniprot) and full-length heavy chain of MYH2 (Uniprot: Q9UKX2). In both constructs, eGFP was fused to the N-terminus. Virus was generated, purified using Vivapure AdenoPACK 100 (Sartorius), and titered as described previously (21). Average titers were 2 × 10^8^ pfu/mL.

C2C12 myoblasts were seeded at the same cell density (1 × 10^5^ cells/mL) on coverslips coated with laminin-1 (MilliporeSigma). Twenty-four hours after seeding, cells were infected with 5 μL of eGFP-MHC adenovirus together with 5 μL of mCherry-UNC45b Co-Chaperone adenovirus (equivalent to MOIs of ~10) (21). The addition of this cochaperone improved sarcomere incorporation of eGFP-MYH2 and MYH7. After 24 hours, the growth medium was exchanged for differentiation medium, and the cells were incubated at 37°C for 5 days to allow for differentiation into skeletal muscle myotubes. The cells were then fixed with 2% PFA for 20 minutes at room temperature as described (21).

### Immunostaining and microscopy of eGFP-MHC–expressing myotubes.

Cells were stained with DAPI (Molecular Probes) to visualize the nucleus. Images of eGFP-myosin–expressing cells were obtained using a Zeiss Airyscan Confocal, using fast Airyscan, 2× Nyquist sampling, and a ×x40 objective (N.A. 1.4) to perform an analysis of sarcomere organization. The same laser settings were used to capture all the images.

The images of eGFP-MHC in skeletal myotubes were analyzed using ImageJ (NIH) to assess sarcomeric incorporation. Lines of a fixed width were drawn along a single sarcomere for 1–4 sarcomeres in the same myofibril, and the intensity profile along the line was measured using the plot profile function. Intensity profiles were imported into an Excel spreadsheet and manually aligned to the minimum value in the center of the sarcomere for each sarcomeric measurement. The intensity plots for each sarcomere were normalized using the normalization function in Prism 10 (GraphPad) and averaged for each myofibril; this data set is available in the supplemental spreadsheet with Supporting Data Values. The average values for each myofibril, for each WT or mutant construct were then combined to generate an average intensity plot across the sarcomere as shown in Supplemental Figure 2C. The distance between peak values, on either side of the M-band, was determined from the mean sarcomere profiles for each myofibril (after normalization) and used to generate the peak-to-peak analysis in Supplemental Figure 2D. Values for a minimum of 20 sarcomeres, from at least 8 myofibrils, were measured.

### Patients.

Muscle biopsy specimens were obtained from patients diagnosed with skeletal myosinopathies and with either *MYH7* or *MYH2* mutations; they were also obtained from age-matched controls with no history of neuromuscular diseases. Details of all the patients are given in Supplemental Table 1. All samples were flash frozen and stored at –80°C until analyzed.

### Solutions.

Relaxing and activating solutions contained 4 mM Mg-ATP, 1 mM free Mg^2+^, 20 mM imidazole, 7 mM EGTA, 14.5 mM creatine phosphate, and KCl to adjust the ionic strength to 180 mM and pH to 7. Additionally, the concentrations of free Ca^2+^ ranged from 1 × 10^–9^ M (relaxing solution, pCa 9) to 1 × 10^–4.5^ M (maximum activating solution, pCa 4.5). The rigor buffer for Mant-ATP chase experiments contained 120 mM K acetate, 5 mM Mg acetate, 2.5 mM K_2_HPO_4_, 50 mM MOPS, and 2 mM DTT with a pH of 6.8 (51).

### Muscle preparation and fiber permeabilization.

Cryopreserved muscle samples were immersed in a membrane-permeabilizing solution (relaxing solution containing glycerol; 50:50 v/v) for 24 hours at –20°C, after which they were transferred to 4°C and bundles of approximately 50 muscle fibers were dissected free and then tied with surgical silk to glass capillary tubes at slightly stretched lengths. These bundles were kept in the membrane-permeabilizing solution at 4°C for an additional 24 hours (to allow a proper skinning process). After these steps, bundles were stored at –20°C for use up to 1 week (51).

### X-ray diffraction recordings and analyses.

On the day of the experiments, bundles were placed in a plastic dish containing the relaxing solution. They were then transferred to the specimen chamber, filled with the relaxing buffer. The ends of these thin-muscle bundles were then clamped at a resting sarcomere length (approximately 2.20 μm). Subsequently, x-ray diffraction patterns were recorded at 15°C using a CMOS camera (Model C11440-22CU, Hamamatsu Photonics, 2,048 × 2,048 pixels) in combination with a 4-inch image intensifier (Model V7739PMOD, Hamamatsu Photonics). The exposure time was 500 ms. The x-ray wavelength was 0.10 nm and the specimen-to-detector distance was 2.14 m. For each preparation, approximately 20–30 diffraction patterns were recorded at the BL40XU beamline of SPring-8 and were analyzed as described previously (52). To minimize radiation damage, the exposure time was kept low and the specimen chamber was moved by 100.00 μm after each exposure. Following x-ray recordings, background scattering was subtracted, and the equatorial reflections as well as the major myosin meridional reflection intensities/spacing were determined as described elsewhere previously (52, 53).

### Mant-ATP chase experiments.

On the day of the experiments, bundles were transferred to the relaxing solution, and single myofibers were isolated. Their ends were individually clamped to half-split copper meshes designed for EM (SPI G100 2010C-XA, width, 3.00 mm), which had been glued to glass slides (Academy, 26.00 × 76.00 mm, thickness 1.00–1.20 mm). Coverslips were then attached to the top (using double-sided tape) to create flow chambers (Menzel-Gläser, 22.00 × 22.00 mm, thickness 0.13–0.16 mm) (51). Subsequently, at 25°C, for each muscle fiber, the sarcomere length was checked using the bright-field mode of a Zeiss Axio Scope A1 microscope. One sarcomere length (approximately 2.20 μm) was used in the present study and further subjected to the Mant-ATP chase protocol. Similar to previous studies (29, 51, 54), each fiber was first incubated for 5 minutes with a rigor buffer. A solution containing the rigor buffer with 250 μM Mant-ATP was then flushed and kept in the chamber for 5 minutes. At the end of this step, another solution made of the rigor buffer with 4.00 mM ATP was added with simultaneous acquisition of the Mant-ATP chase. For fluorescence acquisition, a Zeiss Axio Scope A1 microscope was used with a Plan-Apochromat 20×/0.8 objective and a Zeiss AxioCam ICm1 camera. Frames were acquired every 5 seconds with a 20 ms acquisition/exposure time using a DAPI filter set; images were collected for 5 minutes (tests were run prior to starting the current study where images were collected for 15 minutes instead of 5 minutes, and these tests did not reveal any significant difference in the parameters calculated). Three regions of each individual myofiber were sampled for fluorescence decay using the ROI manager in ImageJ as previously published (29, 51, 54). The mean background fluorescence intensity was subtracted from the average of the fiber fluorescence intensity (for each image taken). Each time point was then normalized by the fluorescence intensity of the final Mant-ATP image before washout (T = 0). These data were then fit to an unconstrained double exponential decay using SigmaPlot 14.0 (Systat Software Inc): Normalized Fluorescence = 1 − P1 (1 − exp^[−t/T1]^) − P2 (1 − exp^[−t/T2]^), where P1 is the amplitude of the initial rapid decay approximating the DRX state with T1 as the time constant for this decay. P2 is the slower second decay approximating the proportion of myosin heads in the SRX with its associated time constant T2 (29, 51, 54).

### Single muscle fiber contractility.

As for Mant-ATP experiments, individual myofibers were dissected in the relaxing solution. They were then individually attached between connectors leading to a force transducer (model 400A; Aurora Scientific) and a lever arm system (model 308B; Aurora Scientific). Sarcomere length was set to approximately 2.50 μm, and the temperature was set to 15°C (51). Since the baths had glass bottom and right angle prisms, fiber cross-sectional areas (CSA) could be estimated from the width and depth, assuming an elliptical circumference. To determine the maximal and submaximal isometric force–generating capacity, myofibers were sequentially bathed in activating buffers with increasing [Ca^2+^] termed pCa. Specific force corresponded to absolute force normalized to myofiber CSA. Submaximal force values were normalized to the maximal force, and the obtained force-pCa curve were fit to the Hill equation (4 parameters). Ca^2+^ sensitivity was then obtained and corresponded to the pCa at which 50% of maximal force is reached. Hill coefficient (*n*H) was also calculated and represented the steepness of the force-pCa curve indicative of the degree of actin-myosin cross-bridge cooperativity (51).

### Immunofluorescence staining and confocal imaging.

To avoid any potential misinterpretation due to the type of MHC (*MYH7*, β-cardiac/skeletal slow MHC; *MYH2*, skeletal fast MHC 2A), for all the above experiments in individually isolated muscle fibers, we assessed the subtype right after the Mant-ATP chase experiments or contractile measurements using immunofluorescence staining and confocal imaging as previously described (51). Briefly, the “used” individual myofibers were individually mounted once again as for the Mant-ATP chase experiments and incubated with A4.951 (1:25, Mouse IgG1, DSHB) or SC71-S (1:25, Mouse IgG1, DSHB) followed by Alexa Fluor 488 or 647 (1:500, goat anti-mouse, Thermo Fisher Scientific). Images were acquired using a confocal microscope (Zeiss Axiovert 200, objectives ×20, ×40, and ×100) equipped with a CARV II confocal imager (BD Biosciences) (51).

### Myofilament model and simulation.

A detailed description of the model and simulations performed is presented in Supplemental Table 2.

### Statistics.

Data are presented as means ± SD. Graphs were prepared and data were analyzed using Prism 10.0 (GraphPad). Statistical significance was set to *P* < 0.05. Control data points were pooled, since no significant differences were observed among them for both in vitro and in vivo experiments. One-way ANOVA with Dunnett’s test post hoc correction was used to compare patients and mutation data against the pooled controls (55).

### Study approval.

All tissue was consented, stored, and used in accordance with the Human Tissue Act, United Kingdom, under local ethical approval (REC 13/NE/0373).

### Data availability.

All the Supporting data values presented in the figures are summarized in Supplemental Tables 3 and 4 and in the Supporting Data Values file. Additionally, if needed, any supporting analytic codes can be made available upon request.

## Author contributions

GC, SK, KSC, MP, and JO designed the work; GC, AH, SK, HFD, FM, ADA, PVDB, CF, NBR, EM, EP, JJV, AO, SP, KO, MGP, EZ, KSC, HI, MP, and JO performed data collection; GC, AH, SK, HFD, KSC, HI, MP, and JO ran data analysis and interpretation; GC, MP, and JO drafted the article; GC, AH, SK, HFD, FM, ADA, PVDB, CF, NBR, EM, EP, JJV, AO, SP, KO, MGP, EZ, KSC, HI, MP, and JO critically revised the article; and GC, AH, SK, HFD, FM, ADA, PVDB, CF, NBR, EM, EP, JJV, AO, SP, KO, MGP, EZ, KSC, HI, MP, and JO approved the final version.

## Supplementary Material

Supplemental data

Supporting data values

## Version 1 10/03/2023

In-Press Preview

## Version 2 11/08/2023

Electronic publication
