# Supplementary material for: Human skeletal myopathy myosin mutations disrupt myosin head sequestration
Source: JCI Insight. 2023 Nov 8;8(21):e172322. doi: 10.1172/jci.insight.172322 (PMC10721271; doi:10.1172/jci.insight.172322)
Supplement: Supplemental data [file jciinsight-8-172322-s120.pdf]

Supplementary information for:

Human Skeletal myopathy myosin mutations disrupt myosin head sequestration

Glenn Carrington, Abbi Hau, Sarah Kosta, Hannah F. Dugdale, Francesco Muntoni, Adele D'Amico, Peter Van den Bergh, Norma B. Romero, Edoardo Malfatti, Juan Jesus Vilchez, Anders Oldfors, Sander Pajusalu, Katrin Õunap, Marta Giralt-Pujol, Edmar Zanoteli, Kenneth S. Campbell, Hiroyuki Iwamoto, Michelle Peckham*, Julien Ochala*

*Corresponding authors: julien.ochala@sund.ku.dk and M.Peckham@leeds.ac.uk

Table S1
Patient and control muscle biopsy samples used.

Age (years)	Gender (M/F)	Mutation	Amino acid change	Heptad position	Disease	Source
			MYH7			
15	F	c.4319delTGC	p.A1440del	*b*	Laing Distal Myopathy	London, UK
16	F	c.4475T>C	p.L1492P	*e*	Laing Distal Myopathy	Rome, Italy
26	M	c.4520-15_4520-9del	p.E1507del	*f*	Congenital Myopathy	Paris, France
68	F	c.4522_4524del	p.E1508del	*g*	Laing Distal Myopathy	Brussels, Belgium
44	F	c.4828G>A	p.E1610K	*c*	Congenital Myopathy	Paris, France
48	F	c.4906G>C	p.A1636P	*g*	Laing Distal Myopathy	Valencia, Spain
49	F	c. c.5005_5007delGAG	p.E1669del	*f*	Laing Distal Myopathy	Gothenburg, Sweden
31	F	c.5292_5294delAGA	p.K1729del	*c*	Laing Distal Myopathy	Valencia, Spain
42	M	c.5292_5294delAGA	p.K1729del	*c*	Laing Distal Myopathy	Valencia, Spain
48	F	c.5292_5294delAGA	p.K1729del	*c*	Laing Distal Myopathy	Valencia, Spain
57	M	c.5292_5294delAGA	p.K1729del	*c*	Laing Distal Myopathy	Valencia, Spain
60	F	c.5292_5294delAGA	p.K1729del	*c*	Laing Distal Myopathy	Valencia, Spain
60	F	c.5292_5294delAGA	p.K1729del	*c*	Laing Distal Myopathy	Valencia, Spain
62	F	c.5292_5294delAGA	p.K1729del	*c*	Laing Distal Myopathy	Valencia, Spain
65	F	c.5292_5294delAGA	p.K1729del	*c*	Laing Distal Myopathy	Valencia, Spain
74	M	c.5292_5294delAGA	p.K1729del	*c*	Laing Distal Myopathy	Valencia, Spain
57	M	c.5533C>T	p.R1845W	*f*	Myosin Storage Myopathy	Valencia, Spain
			MYH2			
1	M	c.5609T>C	p.L1870P	*d*	Congenital Fibre Type Disproportion	Tartu, Estonia
			Controls			
10	M	-	-		-	Sao Paulo, Brazil
20	M	-	-		-	London, UK
20	F	-	-		-	Sao Paulo, Brazil
25	F	-	-		-	London, UK
32	M	-	-		-	Copenhagen, DK
54	F	-	-		-	Copenhagen, DK
71	F	-	-		-	Copenhagen, DK

Table S2

The FiberSim 2.1.0 software has been thoroughly described (1). Briefly, FiberSim tracks the position and status of actin and myosin molecules within a network of compliant thick and thin filaments. For all the simulations, the half-sarcomere lattice was composed of 100 thick filaments and 200 thin filaments. These filaments were arranged in a hexagonal lattice to mimic the architecture of human myofibres. Filaments located at the edge of the lattice were "mirrored" on the opposite side to minimize edge effects (2). Each thin filament was composed of two actin strands. Each strand contained 27 regulatory units, and each regulatory unit contained 7 binding sites. Each thick filament was composed of 54 myosin crowns with each crown consisting of 3 pairs of myosin dimers. Each binding site on actin could be in an inactive (unavailable for myosin binding) or active (available for myosin binding) state. All 7 binding sites from a regulatory unit switched simultaneously between those two states, depending on the Ca^{2+} concentration and on the transition rate constants k_{on} and k_{off} which we set. A cooperative mechanism was also implemented such that the transition probability for a regulatory unit was influenced by the states of its neighbours. Finally, a regulatory unit was prevented from deactivating if one or more myosin head(s) were bound. Although this model only has two explicit thin filament states, it mimics an important feature of the three state thin filament model described by McKillop and Geeves (3) in that bound myosin heads inhibited relaxation. Heads in SRX switched to DRX at a rate k_1 that is assumed to be force-dependent (1). DRX heads could then attach to available binding sites on actin. The attachment and detachment rates depended on x, where x is the distance to the binding site measured parallel to the filaments. The rate functions are provided in Table S2. The detachment rate function (k_4) was updated for the present study so that it had an exponential strain-dependence similar to that measured for single myosin heads via optical trapping (4). Model parameters were chosen to reproduce physiological values for: maximal isometric force (≈ 150-200 kPa at a sarcomere length of 2.2 µm), passive force (≈ 1-2% of the maximal isometric force) and Ca^{2+} sensitivity (pCa_{50} ≈ 5.7). Here are the parameters used.

**Actin kinetics**	**Parameters**
$k_{activate} = [Ca^{2+}] . k_{on} . (1+ n . \gamma_{coop})$	$k_{on} = 2. 10^{7}$ M . s^{-1}<br>$\gamma_{coop} = 10$<br>n = 0,1 or 2 (# of active neighboring RUs)
$k_{deactivate} = k_{off} . (1 + [2 - n] . \gamma_{coop})$	$k_{off} = 100\ s^{-1}$
**Myosin kinetics**	
$k_1 = k_{1,0} + k_{1,f}$. node force	$k_{1,0} = \begin{cases} 45\ s^{-1} \text{ for control} \\ 100\ s^{-1} \text{ for mutation} \end{cases}$<br>$k_{1,f} = 100$ nN s^{-1}
k_2 = constant	$k_2 = 100\ s^{-1}$
$k_3 = k_{3,0} \exp(-\frac{k_{cb} x^2}{2 k_B T})$	$k_{3,0} = 25\ s^{-1}$<br>$k_{cb} = 10^{-3}$ nN.nm^{-1}<br>T = 310 K
$k_4 = \begin{cases} k_{4,0} \exp(-k_{4,1}(x + x_{ps})) \text{ if } x < x_{wall} \\ 1000 \text{ if } x > x_{wall} \end{cases}$	$k_{4,0}=150\ s^{-1}$<br>$k_{4,1}= 0.25\ nm^{-1}$<br>x_{ps} = 5 nm<br>x_{wall} = 8 nm

Table S3
Summary of the results from Figures 2 to 5. Mean values are presented (ND – not done, NF – no filaments).

**Construct/mutation**	**Helicity (%)**	**Tm (°C)**	**Length (nm) Homozygous (heterozygous)**	**Width (nm) homozygous (heterozygous)**	**Peak-Peak GFP-MHC (μm)**
MYH7 WT	100	47.0	313	18	0.98
A1440del	95	49.5	330 (520)	21 (20)	0.95
L1492P	70	41.6	NF (328)	NF (21)	0.83
E1507del	89	46.9	377 (507)	24 (22)	ND
E1508del	94	45.5	234 (370)	22 (18)	0.83
E1610K	102	51.3	566 (585)	23 (14)	0.77
A1636P	83	46.4	NF (185)	NF (23)	ND
E1669del	85	46.7	430 (355)	22 (22)	0.76
K1729del	82	44.7	297 (350)	29 (21)	0.81
R1845W	93	50.3	494 (701)	25 (20)	0.82
MYH2 WT	100	48.3	380	19 (10)	0.64
L1870P	84	45.3	215 (370)	19	ND

Table S4
Summary of the results from Figures 6 to 7. Mean values are presented.

Mutation	P1 (%)	P2 (%)	T1 (sec)	T2 (sec)	Specific force (kPa)	pCa_{50}
β/slow myofibres						
Control	47	53	17	81	148	5.80
A1440del	58	42	16	79	158	5.83
L1492P	59	41	14	80	138	5.73
E1507del	52	48	15	89	159	5.76
E1508del	58	42	16	88	148	5.78
E1610K	60	40	16	90	157	5.81
A1636P	59	41	12	84	148	5.84
E1669del	63	37	16	71	139	5.66
K1729del	60	40	15	82	141	5.85
R1845W	59	41	16	77	164	5.89
L1870P	44	56	15	60	155	5.75
Type IIA myofibres						
Control	49	51	15	87	145	5.79
A1440del	52	48	13	84	156	5.76
L1492P	44	56	18	79	142	5.75
E1507del	46	54	14	99	146	5.89
E1508del	43	57	16	83	131	5.87
E1610K	46	53	16	74	129	5.74
A1636P	44	56	13	71	151	5.66
E1669del	42	58	17	97	147	5.89
K1729del	46	54	16	91	145	5.80
R1845W	47	53	15	98	139	5.77
L1870P	64	36	13	90	131	5.79

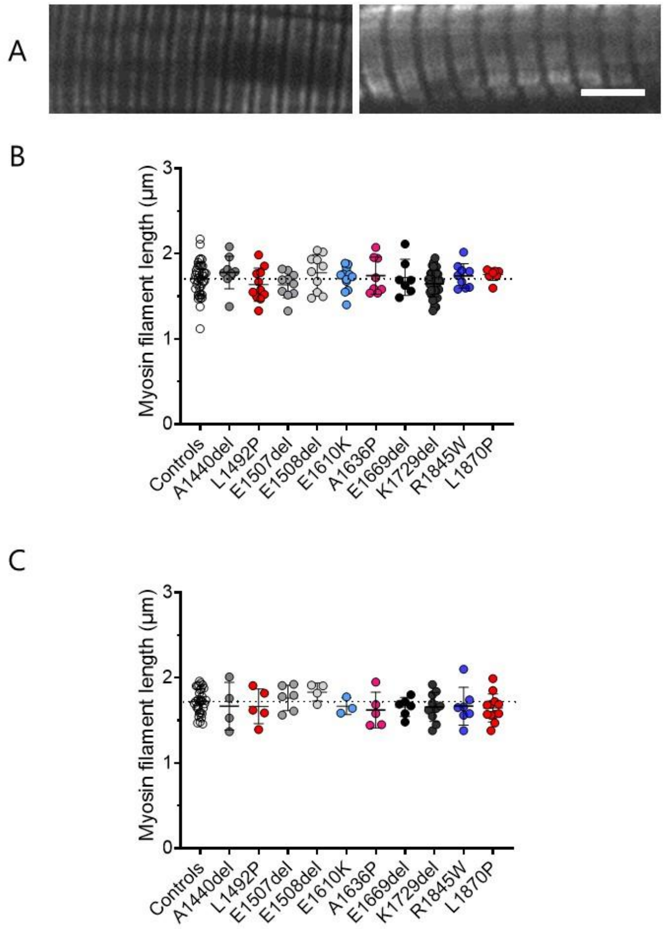

Figure S1

Myosin filament length. Distributed deconvolution (DDecon) was applied from the acquired images with a specific plugin for ImageJ (National Institutes of Health, Bethesda, MD) (5). Note that DDecon is a super-resolution light microscopy technique that allows the computation of filament lengths with a precision of 10.00–20.00 nm (5). All line scans were background-corrected. Distances (and myosin filament lengths) were finally calculated by converting pixel sizes into micrometer using the magnification factor for each image (5). **A.** displays two typical images obtained with confocal microscopy using the A4.951 antibody and allowing the measurement of myosin filament lengths (scale bar: 5 µm). **B.** shows measurements for individual myofibres expressing the β/slow myosin heavy chain isoform in every single subject (n=161) whilst **C** has data relating to muscle fibres expressing the type IIA myosin heavy chain isoform (n=94). Means and standard deviations also appear on these graphs. The one-way ANOVA with Dunnett's test post-correction was used but no significant differences were seen.

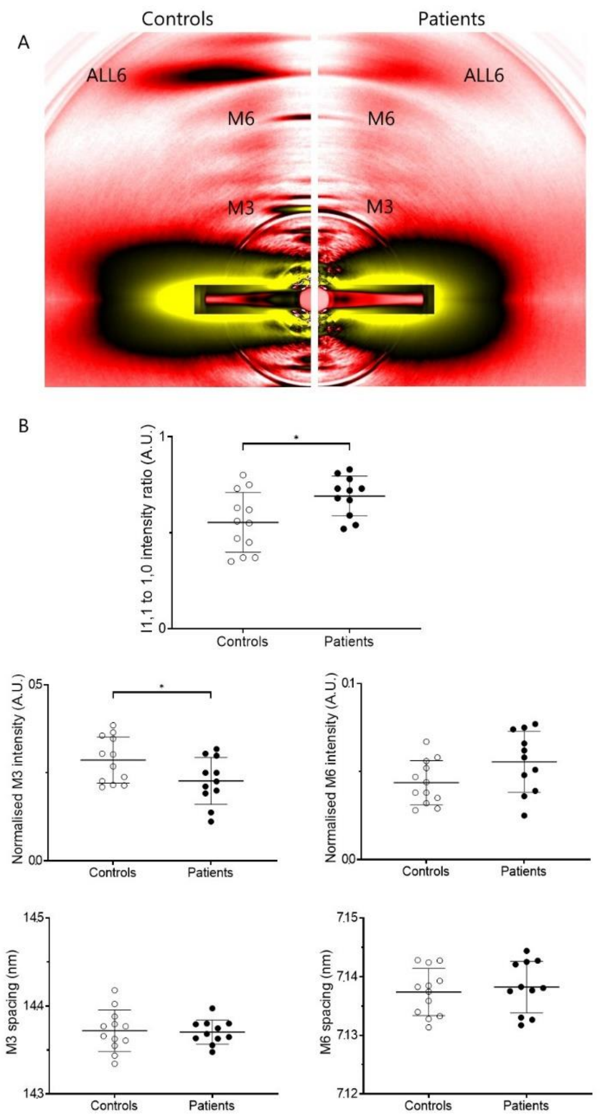

Figure S2

Myosin head order. A. depicts typical X-ray diffraction patterns. **B.** shows equatorial intensity ratio (IR) and the main myosin meridional reflections, namely M3 and M6 (M3 and M6 intensities were normalised to the 6th actin-layer line, ALL6). To ensure reliable results and avoid misinterpretation, we pooled all the patients' data (n=11) together and compared these to images acquired for controls (n=12). Means and standard deviations also appear on these graphs. * The one-way ANOVA with Dunnett's test post-correction was used with $p < 0.05$.

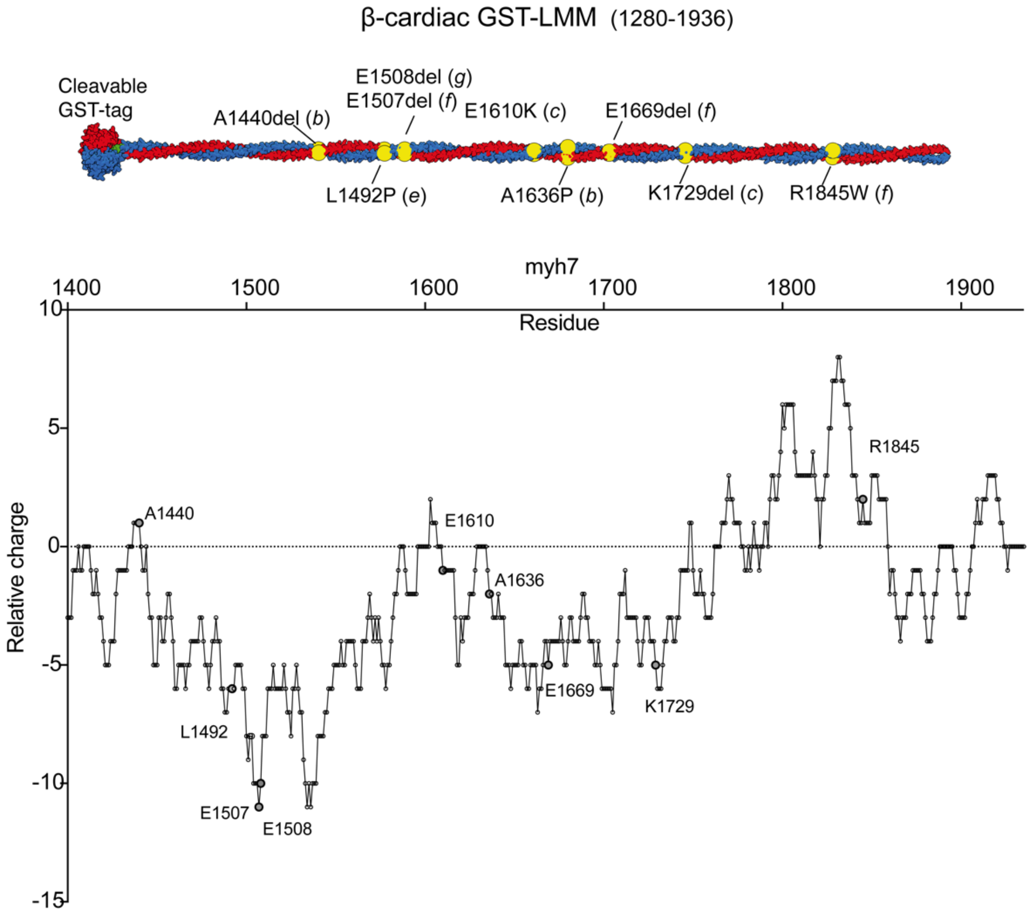

<u>Figure S3</u>
Charge plot of MYH7 LMM region. The diagram shows the positions of mutations in GST-LMM, and the plot shows the alternating regions of positive and negative charges important for filament formation, together with the positions of the mutations.

Figure S4

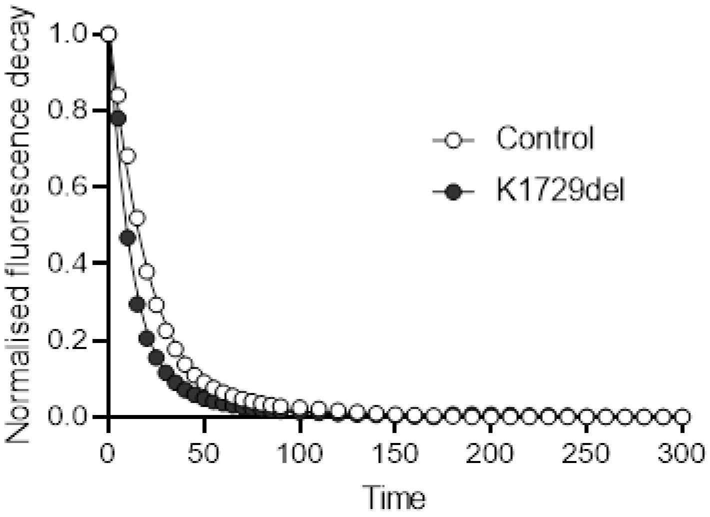

Figure S4
Fluorescent decay with Mant-ATP. Typical Mant-ATP chase experimental data show exponential decays for muscle fibres from one control and one patient (K1729del).

References

1. Kosta S, Colli D, Ye Q, and Campbell KS. FiberSim: A flexible open-source model of myofilament-level contraction. *Biophys J.* 2022;121(2):175-82.
2. Tanner BC, Daniel TL, and Regnier M. Sarcomere lattice geometry influences cooperative myosin binding in muscle. *PLoS Comput Biol.* 2007;3(7):e115.
3. McKillop DF, and Geeves MA. Regulation of the interaction between actin and myosin subfragment 1: evidence for three states of the thin filament. *Biophys J.* 1993;65(2):693-701.
4. Veigel C, Molloy JE, Schmitz S, and Kendrick-Jones J. Load-dependent kinetics of force production by smooth muscle myosin measured with optical tweezers. *Nat Cell Biol.* 2003;5(11):980-6.
5. Ochala J, Gokhin DS, Penisson-Besnier I, Quijano-Roy S, Monnier N, Lunardi J, et al. Congenital myopathy-causing tropomyosin mutations induce thin filament dysfunction via distinct physiological mechanisms. *Hum Mol Genet.* 2012;21(20):4473-85.
